# Author Correction: Selenium-alloyed tellurium oxide for amorphous p-channel transistors

**DOI:** 10.1038/s41586-025-09667-8

**Published:** 2025-09-29

**Authors:** Ao Liu, Yong-Sung Kim, Min Gyu Kim, Youjin Reo, Taoyu Zou, Taesu Choi, Sai Bai, Huihui Zhu, Yong-Young Noh

**Affiliations:** 1 Institute of Fundamental and Frontier Sciences, University of Electronic Science and Technology of China, Chengdu, China; 2 Department of Chemical Engineering, Pohang University of Science and Technology, Pohang, Republic of Korea; 3 Department of Chemistry, Northwestern University, Evanston, IL USA; 4 Korea Research Institute of Standards and Science, Daejeon, Republic of Korea; 5 Department of Nano Science, University of Science and Technology, Daejeon, Republic of Korea; 6 Beamline Research Division, Pohang Accelerator Laboratory, Pohang University of Science and Technology, Pohang, Republic of Korea; 7 School of Physics, University of Electronic Science and Technology of China, Chengdu, China

**Keywords:** Electronic devices, Electrical and electronic engineering

Correction to: *Nature* 10.1038/s41586-024-07360-w Published online 10 April 2024

In the version of the article initially published, in Fig. 3f, which presents the transfer curves of 80 TFTs, two curves were inadvertently duplicated due to a plotting error. The HTML and PDF versions of the article have now been updated with the correct Fig. 3f, as seen in Fig. 1, and we have added the source data for these transfer curves to the article. For completion, we have also added the source data for the batch uniformity plot that is presented in the transparent peer review file. These changes do not affect the statistical analysis or conclusions in the paper.

**Fig. 1:**
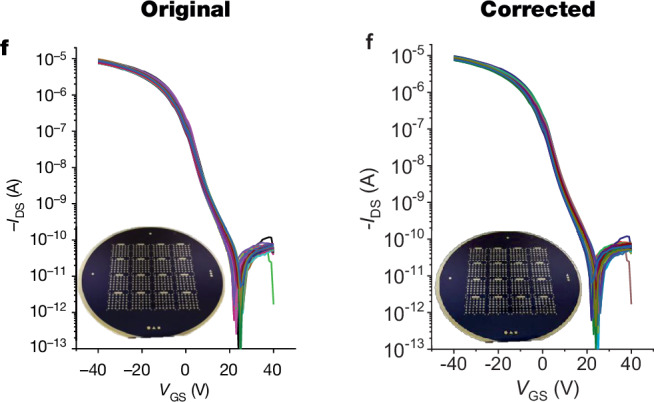
Original and corrected Fig. 3f .

