# Supplementary material for: Selenium-alloyed tellurium oxide for amorphous p-channel transistors
Source: Nature. 2024 Apr 10;629(8013):798–802. doi: 10.1038/s41586-024-07360-w (PMC11111403; doi:10.1038/s41586-024-07360-w)
Supplement: Supplementary file 1 — Supplementary Information [file 41586_2024_7360_MOESM1_ESM.pdf]

nature portfolio

https://doi.org/10.1038/s41586-024-07360-w

Supplementary information

Selenium-alloyed tellurium oxide for amorphous p-channel transistors

In the format provided by the authors and unedited

Supplementary information for

Selenium alloyed tellurium oxide for amorphous p-channel transistors

Ao Liu[1,2,3]*, Yong-Sung Kim[4,5], Min Gyu Kim[6], Youjin Reo[2], Taoyu Zou[2], Taesu Choi[2], Sai Bai[1], Huihui Zhu[2,3,7]*, Yong-Young Noh[2]*

[1]Institute of Fundamental and Frontier Sciences, University of Electronic Science and Technology of China, Chengdu 611731, China

[2]Department of Chemical Engineering, Pohang University of Science and Technology, Pohang, Gyeongbuk 37673, Republic of Korea

[3]Department of Chemistry, Northwestern University, Evanston, Illinois 60208, United States

[4]Korea Research Institute of Standards and Science, Daejeon 34113, Republic of Korea

[5]Department of Nano Science, University of Science and Technology, Daejeon 34113, Republic of Korea

[6]Beamline Research Division, Pohang Accelerator Laboratory, Pohang University of Science and Technology, Pohang, Republic of Korea

[7]School of Physics, University of Electronic Science and Technology of China, Chengdu 611731, China

*A. Liu (ao.liu@uestc.edu.cn), H. H. Zhu (hhzhu@uestc.edu.cn), Y.-Y. Noh (yynoh@postech.ac.kr)

Contents

Supplementary Methods 2

Comprehensive analysis of X-ray absorption spectroscopy (XANES/EXAFS). 2

1) Model structure and reference materials 3

2) Experimental and simulated EXAFS spectra 3

3) Parameters information tables 4

The origin of features in XANES spectra in Fig. 1d 5

The experimental details and the methodology of XANES/EXAFS analysis 5

References 6

Supplementary Methods

Comprehensive analysis of X-ray absorption spectroscopy (XANES/EXAFS).

In this work, we carried out quantitative analysis using the standard EXAFS procedure, and simulations of EXAFS spectra have been performed for the structural analysis. The results show that using three kind of chemical bonds around central Te element for the Se-alloyed Te-TeO_x, Te-O (with oxygen vacancy), Te-Se, and Te-Te, the structural model is well-fitted after EXAFS simulations (**Supplementary Figs. 1a and 1b**).

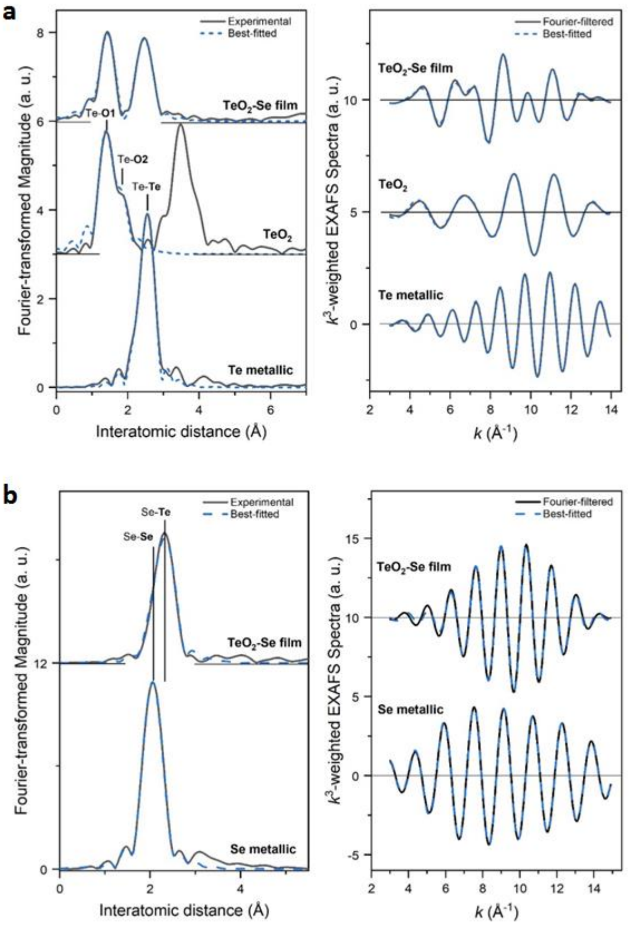

Supplementary Fig. 1 | Analysis of X-ray absorption spectroscopy. (a) (left) Experimental (black solid line) and best-fitted FT features (blue dash line) for the Se-alloyed Te-TeO_x, and (right) corresponding inverse Fourier-transformed Te K-edge EXAFS spectra after curve-fitting process, and reference materials of metallic Te and oxide TeO_2. (b) (left) Experimental (black solid line) and best-fitted FT features (blue dash line) for the Se-alloyed Te-TeO_x, and (right) corresponding inverse Fourier-transformed Se K-edge EXAFS spectra after curve-fitting process, and reference material of metallic Se powder.

More analysis details are provided below, including 1) Model structures; 2) Experimental and simulated EXAFS spectra; 3) Parameter tables.

1) Model structure and reference materials: metallic Te, oxide TeO_2, metallic Se, and metallic-like $TeSe_2$.

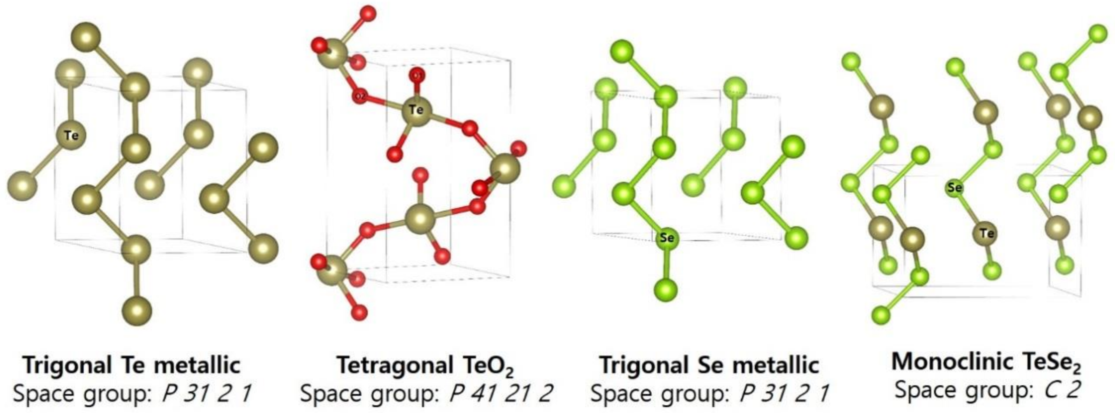

Supplementary Fig. 2 | Reference material models. Chemical model structures of reference materials of Te, TeO_2, Se, and $TeSe_2$.

For possible atomic scatterings in the structural modeling of Se-alloyed Te-TeO_x, the chemical bonds of Te-O (followed by oxygen vacancy), metallic-like Te-Se, and Te-Te have been preliminarily considered for EXAFS simulation. The theoretical single scattering paths have been calculated with reference materials including TeO_2, $TeSe_2$, and metallic Te & Se, respectively. Specifically, for the broad second FT peak around 2.5 Å of the Te K-edge FT and the broad first FT peak around 2.3 Å of the Se K-edge FT, two kinds of chemical bonds, Te-Se/Te-Te and Se-Se/Se-Te, have been introduced, respectively.

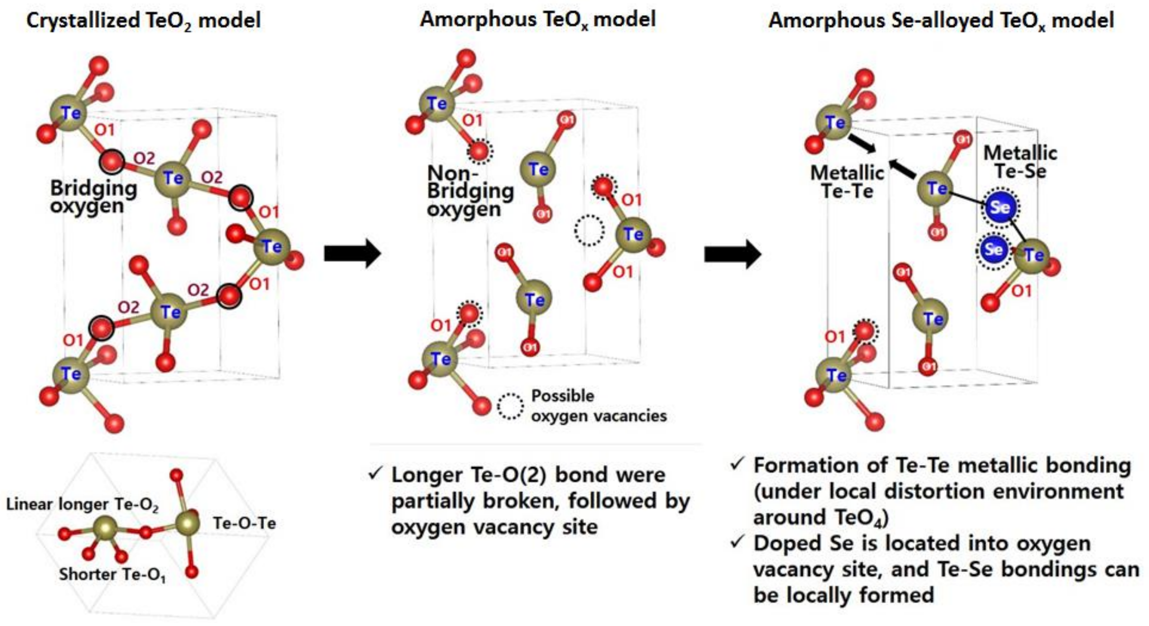

Supplementary Fig. 3 | Different TeO_x models. Local bonding state of crystalized TeO_2, amorphous sub-stoichiometric Te-TeO_x, and amorphous Se-alloyed Te-TeO_x.

Experimental and simulated EXAFS spectra: Based on the proposed structural model above, Supplementary Figs. 1 show the experimental and best-fitted FT features, and corresponding

inverse Fourier-transformed EXAFS spectra after curving-fitting process at Te K-edge and Se K-edge, respectively. Supplementary Tables 1 and 2 present the EXAFS structural parameters for Te K-edge and Se K-edge k^3-weighted EXAFS spectra of Se-alloyed Te-TeO_x sample, respectively. In order to obtain the total amplitude reduction factor, S_0^2, the first shell coordination numbers of metallic Te and TeO_2 are fixed to 2. And each value in parentheses means the uncertainty evaluated from calculation process.

2) Parameters information:

Supplementary Table 1. EXAFS Structural parameters for Te K-edge k^3-weighted EXAFS spectra of Se-alloyed Te-TeO_x sample calculated from EXAFS curve-fitting process.

Sample	Path	Energy shift (eV)	Coordination number	Interatomic distance (Å)	Debye-Waller factor (10^{-3} Å^2)	r-factor of fit**
Te metallic	Te-Te	5.54 (±0.74)	2.00*	2.831 (±0.003)**	5.03 (±0.11)	0.0023
TeO_2	Te-O1	6.45 (±0.85)	2.00*	1.861 (±0.004)	2.22 (±0.26)	0.0057
	Te-O2		2.00*	2.108 (±0.005)	4.15 (±0.47)	
TeO_x:Se Film	Te-O1	4.73 (±1.55)	1.44 (±0.06)	1.878 (±0.006)	3.64 (±0.27)	0.0065
	Te-Se		0.34 (±0.04)	2.576 (±0.010)	2.74 (±0.59)	
	Te-Te		0.84 (±0.06)	2.814 (±0.008)	4.35 (±0.36)	

*In order to obtain the total amplitude reduction factor, S_0^2, the first shell coordination number of metallic Te is fixed to 2. And each value in parentheses means the uncertainty obtained from calculation process.** *R*-factor value which is quality of the fit determined with $\{Re\Delta\chi_k^2+Im\Delta\chi_k^2\}/\{Re(\chi_{kdata})^2+Im(\chi_{kdata})^2\}$, where χ(k) is EXAFS-function) and $\Delta\chi(k)$ means $\chi(k)_{data} - \chi(k)_{best\text{-}fitted}$.

Supplementary Table 2. EXAFS Structural parameters for Se K-edge k^3-weighted EXAFS spectra of Se-alloyed Te-TeO_x sample calculated from EXAFS curve-fitting process.

Sample	Path	Energy shift (eV)	Coordination number	Interatomic distance (Å)	Debye-Waller factor (10^{-3}Å^2)	r-factor of fit***
Se metallic	Se-Se	5.59 (±0.67)	2.00*	2.381 (±0.003)**	4.52 (±0.10)	0.0027
TeO_x:Se Film	Se-Se	7.82 (±1.31)	0.44 (±0.06)	2.394 (±0.010)	2.03 (±0.52)	0.0051
	Se-Te		0.85 (±0.08)	2.605 (±0.008)	2.55 (±0.29)	

*In order to obtain the total amplitude reduction factor, S_0^2, the first shell coordination number of metallic Se is fixed to 2. And each value in parentheses means the uncertainty obtained from calculation process.** *R*-factor value which is quality of the fit determined with $\{Re\Delta\chi_k^2+Im\Delta\chi_k^2\}/\{Re(\chi_{kdata})^2+Im(\chi_{kdata})^2\}$, where χ(k) is EXAFS-function) and $\Delta\chi(k)$ means $\chi(k)_{data} - \chi(k)_{best\text{-}fitted}$.

To enable the clear comparisons and improve the evaluation of data quality, both the original EXAFS spectra and the imaginary part of Fourier Transforms (FTs) are provided in **Supplementary Fig. 4**.

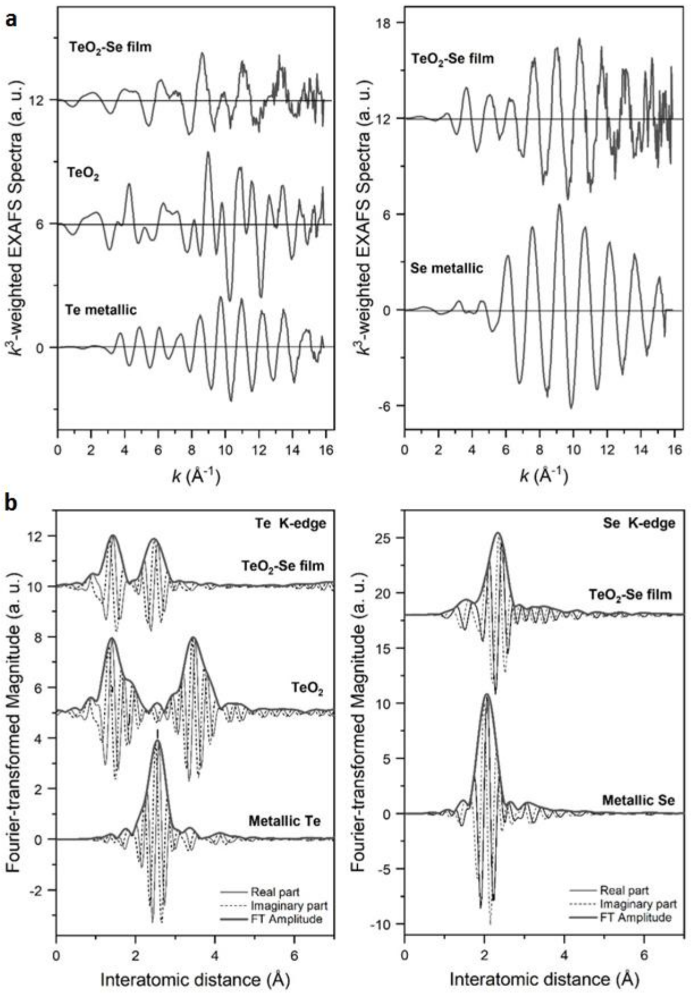

Supplementary Fig. 4 | Original EXAFS spectra as well as the imaginary part of FTs. (a) Experimental k^3-weighted EXAFS spectra and (b) Fourier-transformed magnitudes including real and imaginary parts at the (left) Te K-edge and (right) Se K-edge for the Se-alloyed Te-TeO_x, compared to those of reference Te and Se materials.

The origin of features in XANES spectra in Fig. 1d:

For the reference Te and TeO_2, white line spectral features at ~31820 eV arise from an electric-dipole-allowed transition of the 1*s* electron to the unoccupied 5*p* orbital with the electronic configurations of $[Kr]4d^{10}5s^25p^4$ state and $[Kr]4d^{10}5s^25p^0$ state (Te(IV)), respectively. The Te K-edge XANES feature for the Se-alloyed Te-TeO_x exhibits mixed characteristics resembling both metallic Te and oxide TeO_2 reference materials. Here, we tried to ascertain the chemical composition ratio of these two phases in the Se-alloyed Te-TeO_x using a linear combination of the reference spectra. As a result, we determined an averaged composition ratio of approximately 4:6 for metallic Te and TeO_2 phases.

The experimental details and the methodology of XANES/EXAFS analysis:

X-ray absorption spectroscopy: Te and Se K-edge X-ray absorption spectra of the Se-alloyed Te-TeO_x film, X-ray absorption near edge structure (XANES) and extended X-ray absorption fine structure (EXAFS), were collected on BL10C beam line (using multiple wiggler source) at the

Pohang light source (PLS-II) with top-up mode operation under a ring current of 250 mA at 3.0 GeV. The monochromatic X-ray beam could be obtained using liquid-nitrogen cooled Si(311) double crystal monochromator (Bruker ASC). For Te (31814 eV) and Se (12658 eV) K-edge XAFS measurements, X-ray absorption spectroscopic data were recorded in fluorescence mode with 7 channels silicon drift detector (SDD, Rayspec Ltd.) as photon detector. Higher order harmonic contaminations were eliminated by detuning to reduce the incident X-ray intensity by ~20%. Energy calibration has been carried out with reference Te and Se metal powders.

XAFS data analysis: The XAFS data analysis were performed through the standard XAFS procedure.[1-4)] Using AUTOBK module in UWXAFS package[5)], the k^3-weighted Te K-edge and Se K-edge EXAFS spectra, $k^3\chi(k)$, have been obtained through background removal and normalization processes. The $k^3\chi(k)$ spectra have been Fourier-transformed (FT) in the k ranges between 3.5 and 14.0 $Å^{-1}$ (Te K-edge) and 15.0 $Å^{-1}$ (Se K-edge). The experimental FT spectra have been inversely Fourier-transformed with the *hanning* window function in the r space range between 1.0 and 3.2 Å (Te K-edge) and 3.0 $Å^{-1}$ (Se K-edge). To determine the EXAFS structural parameters for the first bond pairs, the curve-fitting process has been carried out by using the single bonding model. Theoretical single scattering paths of the first shells around central Te element have been calculated with FEFF9 code[6-7)] under the space groups of *P 41 21 2* for the tetragonal TeO_2 model, *P 31 2 1* for the trigonal Te metallic, *C2* for the monoclinic $TeSe_2$, *P 31 2 1* for the trigonal Se metallic. In the EXAFS curve fitting process with FEFFIT module, total amplitude reduction factor, S_0^2, were fixed to 0.7 for the Te K-edge XAFS and 0.9 for the Se K-edge XAFS, which were obtained after EXAFS fitting for metallic Te and Se EXAFS spectra with constant two coordination numbers. The EXAFS structural parameters, interatomic distance (r), coordination numbers (N), Debye-Waller factor (σ^2), have been determined within allowed R-factor value which is quality of the fit with $\{Re\Delta\chi_k^2+Im\Delta\chi_k^2\}/\{Re(\chi_{kdata})^2+Im(\chi_{kdata})^2\}$, where χ(k) is EXAFS-function) and $\Delta\chi$(k) means $\chi(k)_{data}$ - $\chi(k)_{best\text{-}fitted}$. For *k-r* space correlations, *Morlet* wavelet-transformed EXAFS have been also obtained with proper values of η and σ in equation spectra[8-9)] as follows;

$$\psi(t) = \frac{1}{\sqrt{2\pi}\sigma}(e^{i\eta t} - e^{-\eta^2\sigma^2/2})e^{-t^2/2\sigma^2}$$

where the η is the frequency of the oscillation functions and the σ is the half width.

References

1. J. J. Rehr and R. C. Albers, Theoretical Approaches to X-ray Absorption Fine Structure, *Rev. Mod. Phys*. 72, 621, (2000).
2. J.J. Rehr and R.C. Albers,Multiple Scattering theory: Scattering-matrix formulation of curved-wave multiple-scattering theory: Application to x-ray-absorption fine structure, *Phys. Rev. B* 41, 8139 (1990).
3. M. Newville, IFEFFIT: interactive EXAFS analysis and FEFF fitting, *J. Synchrotron Rad*. 8, 322 (2001).
4. ATHENA, ARTEMIS, HEPHAESTUS: data analysis for X-ray absorption spectroscopy using IFEFFIT, B. Ravel and M. Newville, *J. Synchrotron Rad*. 12, 537 (2005).
5. E.A. Stern ∗, M. Newville, B. Ravel, Y. Yacoby, D. Haskel, The UWXAFS analysis package: philosophy and details, *Physica B: Condensed Matter*, 208-209, 117, 1995.

6. J. J. Rehr, J. J. Kas, F. D. Vila, M. P. Prange, K. Jorissen, Parameter-free calculations of X-ray spectra with FEFF9. *Phys. Chem. Chem. Phys*., 12, 5503- 5513, 2010.
7. A.L. Ankudinov, B. Ravel, J.J. Rehr, and S.D. Conradson, FEFF8: Real Space Multiple Scattering Calculation of XANES, *Phys. Rev. B* 58, 7565, 1998.
8. H. Funke*, A. C. Scheinost, Wavelet analysis of extended x-ray absorption fine structure data, *Phys. Rev. B*, 71, 094110, 2005.
9. H. Funke*, M. Chukalina, A. C. Scheinost, A new FEFF-based wavelet for EXAFS data analysis, *J. Synchrotron Rad.* 14, 426-432, 2007.
